# Association of stress hyperglycemia ratio and in-hospital mortality in patients with coronary artery disease: insights from a large cohort study

**DOI:** 10.1186/s12933-022-01645-y

**Published:** 2022-10-19

**Authors:** Wei Xu, Qirui Song, Xiang Wang, Zinan Zhao, Xuyang Meng, Chenxi Xia, Yibo Xie, Chenguang Yang, Ying Guo, Yatong Zhang, Fang Wang

**Affiliations:** 1 Emergency Center, State Key Laboratory of Cardiovascular Disease of China, National Center for Cardiovascular Diseases, National Clinical Research Center of Cardiovascular Diseases, Fuwai Hospital, Chinese Academy of Medical Sciences and Peking Union Medical College, 100037 Beijing, China; 2 Hypertension Center, State Key Laboratory of Cardiovascular Disease of China, National Center for Cardiovascular Diseases of China, Fuwai Hospital, Chinese Academy of Medical Sciences, Peking Union Medical College, 100037 Beijing, China; 3 Department of Cardiology, Beijing Hospital, National Center of Gerontology, Institute of Geriatric Medicine, Chinese Academy of Medical Sciences, Beijing, China; 4 Graduate School of Peking Union Medical College, Chinese Academy of Medical Sciences, Beijing, China; 5 Department of Pharmacy, Institute of Geriatric Medicine, Beijing Hospital, National Center of Gerontology, Chinese Academy of Medical Sciences, Beijing Key Laboratory of Assessment of Clinical Drugs Risk and Individual Application (Beijing Hospital), Beijing, China; 6 Department of Information Center, Beijing Hospital, National Center of Gerontology, Institute of Geriatric Medicine, Chinese Academy of Medical Sciences, Beijing, China

**Keywords:** Coronary artery disease, Diabetes, Mortality, Stress hyperglycemia, Stress hyperglycemia ratio

## Abstract

**Background:**

Stress hyperglycemia is strongly associated with poor clinical outcomes in patients with acute coronary syndrome (ACS). Recently, the stress hyperglycemia ratio (SHR) has been proposed to represent relative hyperglycemia. Studies regarding the relationship between SHR and mortality in coronary artery disease (CAD) are limited. This study aimed to clarify the association between SHR and in-hospital mortality in patients with CAD.

**Methods:**

A total of 19,929 patients with CAD who were hospitalized in Beijing Hospital were enrolled in this study. Patients with an estimated glomerular filtration rate < 30 ml/min, cancer, or missing blood glucose/HbA1c data were excluded; therefore, 8,196 patients were included in the final analysis. The patients were divided into three groups based on tertiles of SHR: T1 group (SHR < 0.725, n = 2,732), T2 group (0.725 ≤ SHR < 0.832, n = 2,730), and T3 group (SHR ≥ 0.832, n = 2,734). The primary endpoint was in-hospital mortality.

**Results:**

The overall in-hospital mortality rate was 0.91% (n = 74). After adjusting for covariates, SHR was significantly associated with in-hospital mortality in patients with CAD [odds ratio (OR) = 17.038; 95% confidence interval (CI) = 9.668–30.027; *P <* 0.001], and the T3 group had a higher risk of in-hospital mortality (OR = 4.901; 95% CI = 2.583–9.297; *P <* 0.001) compared with T1 group. In the subgroup analysis, the T3 group had an increased risk of mortality among patients with pre-diabetes mellitus (pre-DM) (OR = 9.670; 95% CI = 1.886–49.571; *P* = 0.007) and diabetes mellitus (DM) (OR = 5.023; 95% CI = 2.371–10.640; P < 0.001) after adjustments for covariates. The relationship between SHR and in-hospital mortality among patients with ACS and chronic coronary syndrome was consistent with the main finding. SHR and in-hospital mortality exhibited a dose-response relationship, and the risk of in-hospital mortality increased when the SHR index was above 1.20. Moreover, the area under the curve of SHR for predicting in-hospital mortality in patients with CAD was 0.741.

**Conclusion:**

SHR is significantly associated with in-hospital mortality in patients with CAD. SHR may be an effective predictor of in-hospital mortality in patients with CAD, especially for those with pre-DM and DM.

**Supplementary Information:**

The online version contains supplementary material available at 10.1186/s12933-022-01645-y.

## Background

Coronary artery disease (CAD) is the leading cause of mortality worldwide despite the development of prevention and treatment strategies [1, 2]. Metabolic disorders, including hyperglycemia, insulin resistance, and dyslipidemia that result from an unhealthy lifestyle and Western diet, lead to CAD [3]. Chronic hyperglycemia caused by diabetes mellitus (DM) is a well-defined risk factor for adverse cardiovascular events. Stress-induced glucose levels are associated with poor clinical outcomes among patients with acute coronary syndrome (ACS) [4–6], and this association is stronger in patients without DM than in those with DM [4, 7, 8]. Increased glucose levels at the time of hospital admission may be a result of acute stress reactivity or chronic hyperglycemia. Thus, the use of relative hyperglycemia to identify stress-induced hyperglycemia has been suggested [9, 10]. The stress hyperglycemia ratio (SHR) represents relative hyperglycemia and has been proven to be effective in predicting worsening prognosis in patients with severe acute disease [9]. Several studies have reported that SHR was significantly associated with adverse clinical outcomes in patients with ACS [11, 12]. However, this association may be a comprehensive effect of a heterogeneous population with ACS. Therefore, it is necessary to further assess this relationship under specific conditions. Studies regarding the relationship of SHR and mortality in patients with CAD are limited. This study aimed to clarify the association between SHR and in-hospital mortality in patients with CAD.

## Methods

### Study design and population

**Fig. 1:**
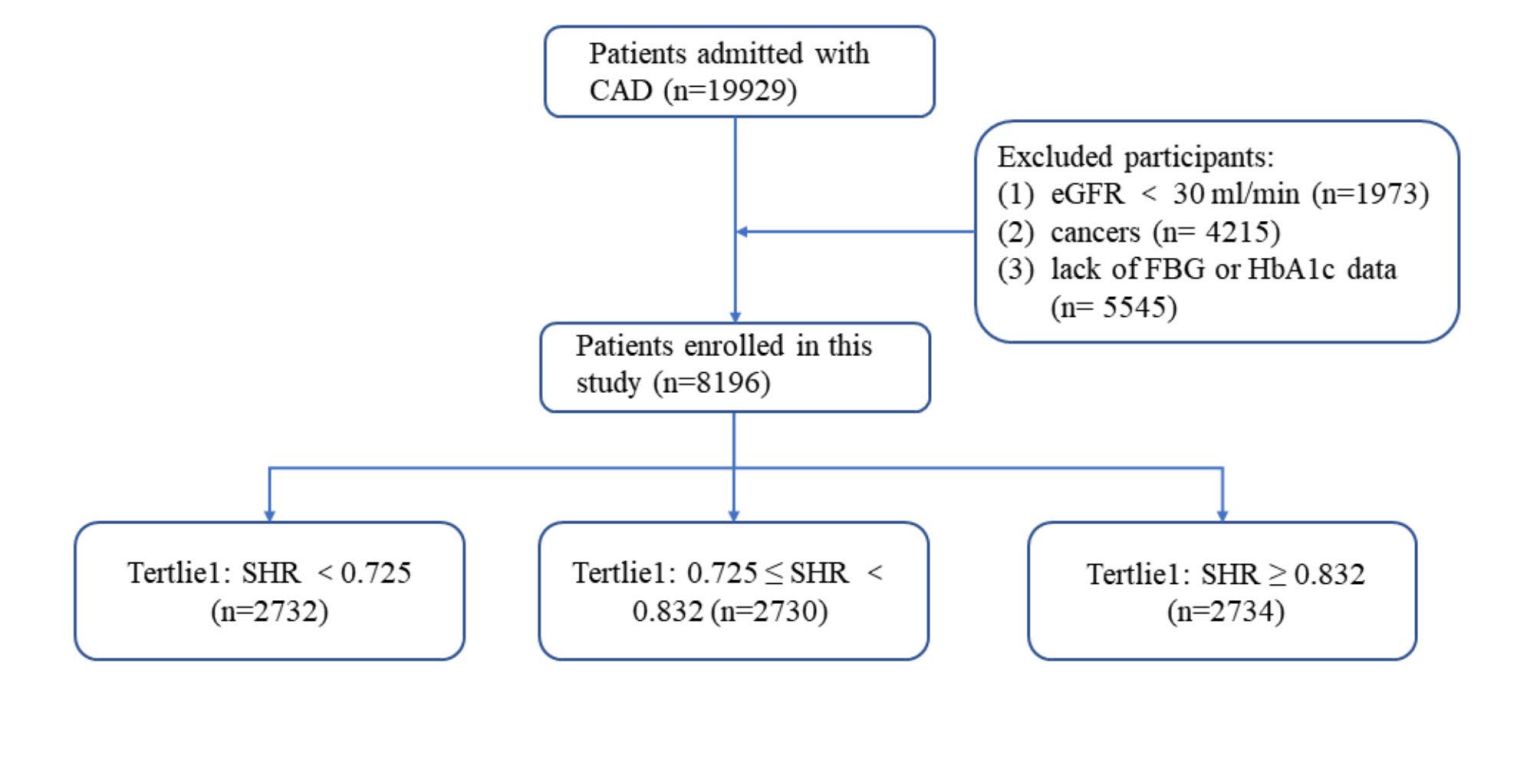
Patient flowchart

This retrospective cohort study was conducted at Beijing Hospital in accordance with the Declaration of Helsinki and was approved by the Ethics Committee of Beijing Hospital. Written informed consent was obtained from all patients. A total of 19,929 patients diagnosed with CAD who were hospitalized at Beijing Hospital between January 1, 2016 and December 30, 2021 were enrolled in this study. However, 1,973 patients with an estimated glomerular filtration rate (eGFR) < 30 ml/min; 4,215 patients with cancer; and 5,545 patients with missing blood glucose or HbA1c data were excluded (Fig. 1). A total of 8,196 patients were included in the final analysis. The patients were divided into three groups according to the tertiles of SHR: T1 group (SHR < 0.725, n = 2,732), T2 group (0.725 ≤ SHR < 0.832, n = 2,730), and T3 group (SHR ≥ 0.832, n = 2,734). The primary endpoint was in-hospital mortality rate.

### Data measurement and definitions

Patient demographics (age, sex, height, weight, smoking status, and drinking status), medical history (hypertension, diabetes mellitus, and cancer), and laboratory test results were obtained from the medical records at Beijing Hospital. Systolic blood pressure (SBP), diastolic blood pressure (DBP), and heart rate (HR) on hospital admission were recorded, as was the patient’s use of antihypertensive, antiplatelet, or antilipidemic medications. In-hospital death was defined as all-cause death that occurred in-hospital within 30 days of admission and was collected via medical records. The admission blood glucose (ABG) referred to the first-measured random serum glucose within the first 24 h of admission. Blood samples for testing HbA1c, total cholesterol (TC), triglycerides (TG), high-density lipoprotein cholesterol (HDL-C), low-density lipoprotein cholesterol (LDL-C), and eGFR were obtained from the cubital vein after at least eight hours of fasting. Blood glucose, eGFR, TC, TG, HDL-C, and LDL-C were measured using a LABOSPECT 008 system (Hitachi, Tokyo, Japan), and the HbA1c level was determined using high-performance liquid chromatography (G8, TOSOH, Tokyo, Japan) at the central laboratory of Beijing Hospital. Body mass index (BMI) was calculated as weight (kg) divided by the squared height (m), and eGFR was calculated according to the Chronic Kidney Disease Epidemiology Collaboration creatinine equation [13].

SHR was calculated using the following equation: SHR = ABG (mmol/L) / [1.59 × HbA1c(%) − 2.59] [9].

CAD was defined as ≥ 50% lumen stenosis in at least one major coronary artery (left anterior descending, left circumflex, or right coronary arteries). Henceforth, diabetes refers to type 2 diabetes and was defined as a history of type 2 diabetes or an HbA1c > 6.5% [14]. Pre-diabetic status was defined as patients without a history of diabetes but with HbA1c ranging from 5.7 to 6.4%. Patients with no history of diabetes or an HbA1c ≤ 5.7% were regarded as normoglycemia (NGR).

### Statistical analysis

Continuous variables are presented as mean ± standard deviation (SD) or median and interquartile range (25–75%), as appropriate. Categorical variables are presented as numbers (n) and percentages (%). A one-way analysis of variance or Kruskal–Wallis test was conducted to compare the baseline variables between the SHR groups. Chi-square tests were performed to compare the categorical variables.

To analyze the association between SHR and in-hospital deaths, odds ratios (OR) and 95% confidence intervals (CI) were calculated using a logistic regression analysis. In the current study, model 1 was unadjusted; model 2 was adjusted for age and sex; and model 3 was further adjusted for BMI, SBP, DBP, eGFR, TC, smoking status, and drinking status. Restricted cubic splines (RCS) were used to examine the shape of the association between SHR and in-hospital mortality. Receiver operating curves (ROC) were used to calculate the area under the curve (AUC) of the SHR for predicting in-hospital mortality in patients with CAD. Further, we also did subgroup analysis according to the ACS and chronic coronary syndromes (CCS) groups and different diabetes status by using logistic regression analysis.

All statistical analyses were performed using SAS version 9.4 (SAS Institute, Inc., Cary, NC) and R language version 4.0.4. Statistical significance was set at *P* < 0.05.

## Results

### Baseline characteristics

The mean patient age was 68 ± 11 years. A total of 5,266 (64.25%) patients were men, and 4412 (53.83%) had DM. Patient age, sex, BMI, HR, DBP, smoking status, drinking status, hypertension, NGR, pre-DM, DM, ACS, glucose, HbA1c, HDL-C, LDL-C, TC, TG, eGFR, use of antiplatelets, and use of antilipidemic drugs were significantly different between the three groups (all, *P* < 0.05) (Table 1).

**Table 1:** Patient baseline characteristics according to tertiles of stress hyperglycemia ratio

	Total (n = 8196)	T1 (n = 2732)	T2 (n = 2730)	T3 (n = 2734)	P-value
Age (years)	68 ± 11	69 ± 10	68 ± 11	67 ± 11	<0.001*
Male (n, %)	5266 (64.25%)	1659 (60.72%)	1726 (63.22%)	1881 (68.80%)	<0.001*
BMI (Kg/m2)	25.57 ± 3.41	25.39 ± 3.42	25.57 ± 3.34	25.72 ± 3.45	<0.001*
HR (beats/min)	78 ± 13	77 ± 13	77 ± 13	79 ± 14	<0.001*
SBP (mmHg)	136 ± 19	136 ± 19	136 ± 18	137 ± 20	0.138
DBP (mmHg)	77 ± 12	76 ± 13	77 ± 12	77 ± 12	<0.001*
Smoking (n, %)	3524 (43.00%)	1113 (40.74%)	1163 (42.60%)	1248 (45.65%)	0.001*
Drinking (n, %)	4672 (57.00%)	1466 (53.66%)	1565 (57.33%)	1641 (60.02%)	<0.001*
Hypertension (n, %)	5894 (71.91%)	1992 (72.91%)	1909 (69.93%)	1993 (72.90%)	0.018*
NGR (n, %)	1395 (17.02%)	149 (5.45%)	567 (20.77%)	679 (24.84%)	<0.001*
Pre-DM (n, %)	2389 (29.15%)	842 (30.82%)	1016 (37.22%)	531 (19.42%)	<0.001*
DM (n, %)	4412 (53.83%)	1741 (63.73%)	1147 (42.01%)	1524 (55.74%)	<0.001*
ACS (n, %)	3001 (36.62%)	906 (33.16%)	1004 (36.78%)	1091 (39.90%)	<0.001*
Glucose (mmol/l)	6.55 ± 2.39	5.52 ± 1.30	5.96 ± 1.32	8.17 ± 3.12	<0.001*
HbA1c (%)	6.80 ± 1.38	7.22 ± 1.53	6.46 ± 1.06	6.71 ± 1.40	<0.001*
HDL-C (mg/dL)	1.06 ± 0.27	1.08 ± 0.28	1.07 ± 0.26	1.03 ± 0.27	<0.001*
LDL-C (mg/dL)	2.17 ± 0.83	2.10 ± 0.80	2.21 ± 0.85	2.21 ± 0.83	<0.001*
TC (mg/dL)	3.81 ± 0.98	3.72 ± 0.95	3.85 ± 0.99	3.87 ± 0.99	<0.001*
TG (mg/dL)	1.43 ± 0.94	1.31 ± 0.82	1.44 ± 0.89	1.57 ± 1.07	<0.001*
eGFR (ml/min)	84.68 ± 17.49	82.98 ± 17.88	86.22 ± 16.04	84.84 ± 18.32	<0.001*
**Medications**					
Antiplatelets (n, %)	6844 (83.60%)	2284 (83.60%)	2318 (84.91%)	2242 (82.00%)	0.015*
Antihypertensive drugs (n, %)	6200 (76.65%)	2086 (76.35%)	2071 (75.86%)	2043 (74.73%)	0.355
Antilipidemic drugs (n, %)	6901 (84.20%)	2306 (84.41%)	2362 (86.52%)	2233 (81.68%)	<0.001*

* Statistically significant P values

Patients in the T3 group were more likely to have higher glucose and HbA1c, dyslipidemia, and a history of DM, hypertension, and a diagnosis of ACS.

### Clinical outcomes

The overall in-hospital mortality rate was 0.91% (n = 74). SHR was significantly associated with the risk of in-hospital deaths (OR = 22.309; 95% CI = 13.194–37.723; *P*<0.001) (Table 2). SHR was identified as an independent risk factor for in-hospital mortality in patients with CAD after adjusting for potential risk factors in model 2 (OR = 20.177; 95% CI = 11.485–35.444; *P* < 0.001) and model 3 (OR = 17.038; 95% CI = 9.668–30.027; *P* < 0.001). Highest value of SHR group (T3 group) was correlated with a higher risk of in-hospital mortality (OR = 4.481; 95% CI = 2.389–8.404; *P* < 0.001) in model 1. After adjusting for age, sex, BMI, SBP, DBP, smoking, drinking, ACS, TC, eGFR, the T3 group had a higher risk of in-hospital mortality by 4.9-fold than the T1 group (OR = 4.901; 95% CI = 2.583–9.297; *P* < 0.001).

**Table 2:** Associations between stress hyperglycemia ratio and in-hospital mortality

	Model 1			Model 2			Model 3		
	OR	95% CI	P-value	OR	95% CI	P-value	OR	95% CI	P-value
SHR index	22.309	13.194–37.723	<0.001	20.177	11.485–35.444	<0.001	17.038	9.668–30.027	<0.001
T1	Reference			Reference			Reference		
T2	0.750	0.315–1.782	0.514	0.913	0.383–2.181	0.838	0.937	0.390–2.246	0.883
T3	4.481	2.389–8.404	<0.001	5.012	2.657–9.453	<0.001	4.901	2.583–9.297	<0.001

Model 1: Original model

Model 2: Adjusted for age and sex

Model 3: Adjusted for age, sex, BMI, SBP, DBP, smoking, drinking, ACS, TC, and eGFR.

**Fig. 2:**
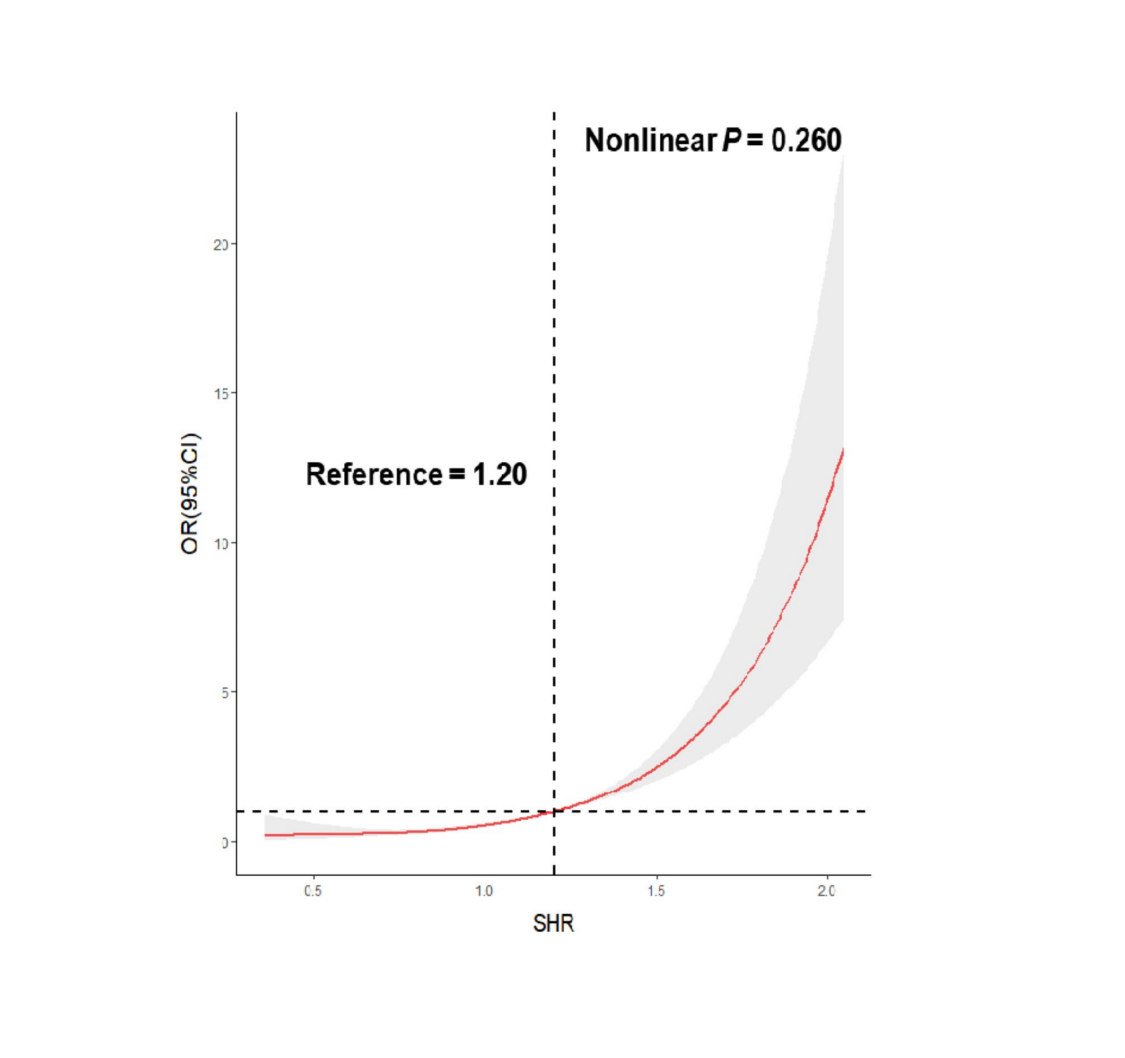
Restricted cubic splines for the odds ratio of in-hospital mortality

**Fig. 3:**
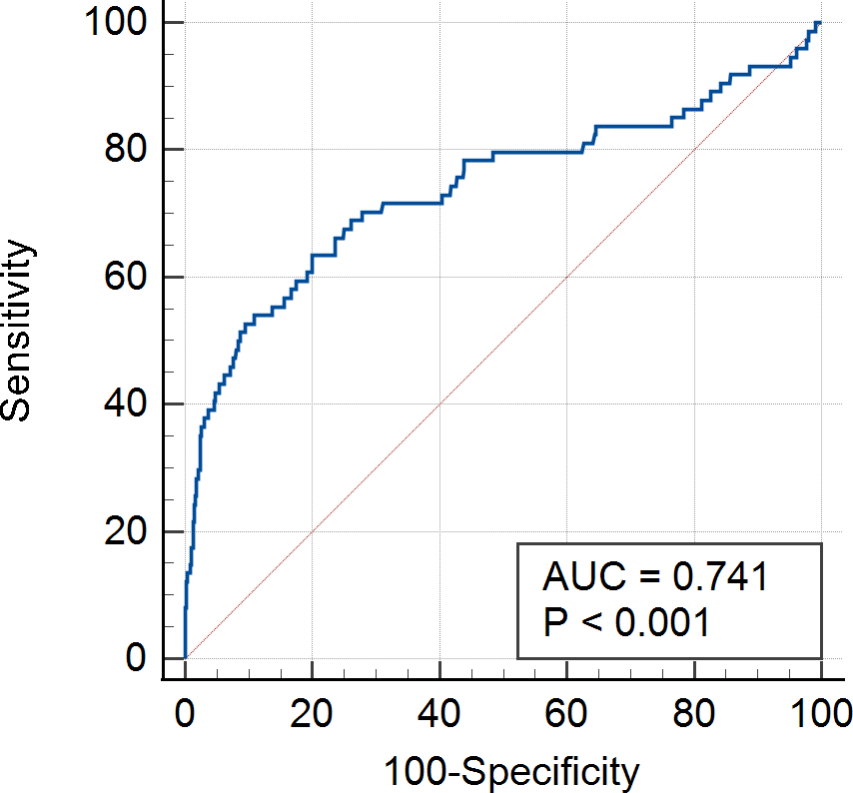
Receiver operating curves of stress hyperglycemia ratio for predicting in-hospital mortality

A dose-response relationship between SHR and in-hospital mortality was observed (nonlinear *P* value = 0.260) (Fig. 2). The ORs of in-hospital mortality significantly increased when the SHR index was above 1.20. The AUC of SHR for predicting in-hospital deaths was 0.741, which is considered a strong predictive ability (Fig. 3). Additionally, both the ABG (AUC = 0.757) and SHR (AUC = 0.741) showed a strong predictive power in the total study population (Supplementary material Figure S1a); however, SHR (AUC = 0.766) was slightly better than ABG (AUC = 0.728) and better than HbA1c (AUC = 0.594) in predicting in-hospital mortality among patients with CAD and diabetes (Figure S1b). In patients with CCS, the AUC of ABG, HbA1c, and SHR was 0.750, 0.563 and 0.694, respectively (Figure S1c). For patients with ACS, the AUC of SHR (AUC = 0.779) for predicting in-hospital mortality was better than ABG (AUC = 0.757) and HbA1c (AUC = 0.528) (Figure S1d).

### Subgroup analyses

Table 3 shows the associations between SHR and in-hospital mortality in patients with ACS and CCS. The multivariable logistic regression model (model 3) illustrated that SHR was an independent predictor for in-hospital mortality in both ACS group (OR = 19.351; 95% CI = 8.397–44.592; *P* <0.001) and CCS group (OR = 15.453; 95% CI = 6.924–34.488; *P* <0.001). Moreover, the T3 group in patients with ACS had a higher risk of in-hospital mortality by 7.9-fold than that of the T1 group (OR = 7.939; 95% CI = 2.723–23.151; *P* <0.001). In those with CCS, the T3 group had a higher risk of in-hospital mortality by 3.6-fold than that of the T1 group (OR = 3.564; 95% CI = 1.567–8.107; *P* = 0.002).

**Table 3:** Associations between stress hyperglycemia ratio and in-hospital mortality in patients with acute coronary syndromes and chronic coronary syndromes

Subgroup	Model 1			Model 2			Model 3		
	OR	95% CI	P-value	OR	95% CI	P-value	OR	95% CI	P-value
ACS									
SHR	19.547	9.008–42.417	<0.001	21.057	9.180-48.299	<0.001	19.351	8.397–44.592	<0.001
T1	Reference			Reference			Reference		
T2	1.129	0.302–4.216	0.857	1.308	0.348–4.911	0.691	1.558	0.410–5.917	0.515
T3	6.158	2.157–17.582	0.001	6.858	2.387–19.702	<0.001	7.939	2.723–23.151	<0.001
CCS									
SHR	23.664	11.547–48.495	<0.001	17.841	8.099–39.299	<0.001	15.453	6.924–34.488	<0.001
T1	Reference			Reference			Reference		
T2	0.528	0.159–1.756	0.298	0.685	0.204–2.303	0.541	0.664	0.197–2.238	0.509
T3	3.369	1.509–7.519	0.003	3.770	1.668–8.521	0.001	3.564	1.567–8.107	0.002

Model 1: Original model

Model 2: Adjusted for age and sex

Model 3: Adjusted for age, sex, BMI, SBP, DBP, smoking, drinking, ACS, TC, and eGFR.

In Table 4, among patients with pre-DM, the univariable logistic regression model (model 1) suggested that the T3 group had a higher risk of in-hospital mortality than the T1 group (OR = 7.241; 95% CI = 1.559–33.645; *P* = 0.012). This higher risk remained in model 2 (OR = 8.073; 95% CI = 1.701–38.315; *P* = 0.009) and model 3 (OR = 9.670; 95% CI = 1.886–49.571; *P* = 0.007). Among patients with CAD and DM, a higher SHR was associated with an increased risk of in-hospital mortality in model 1 (OR = 4.788; 95% CI = 2.304–9.954; *P* < 0.001), model 2 (OR = 4.974; 95% CI = 2.377–10.406; *P* < 0.001), and model 3 (OR = 5.023; 95% CI = 2.371–10.640; *P* < 0.001).

**Table 4:** Associations between stress hyperglycemia ratio and in-hospital mortality in patients with and without diabetes mellitus

Diabetes status	Events/n	Model 1			Model 2			Model 3		
		OR	95% CI	P-value	OR	95% CI	P-value	OR	95% CI	P-value
**NGR**										
T1	1/149	Reference			Reference			Reference		
T2	0/567	-	-	-	-	-	-	-	-	-
T3	7/679	1.542	0.188–12.625	0.687	2.215	0.257–19.111	0.470	1.020	0.071–14.581	0.988
**Pre-DM**										
T1	2/842	Reference			Reference			Reference		
T2	2/1016	0.828	0.116–5.893	0.851	0.996	0.139–7.142	0.997	1.036	0.140–7.685	0.973
T3	9/531	7.241	1.559–33.645	0.012	8.073	1.701–38.315	0.009	9.670	1.886–49.571	0.007
**DM**										
T1	9/1741	Reference			Reference			Reference		
T2	7/1147	1.182	0.439–3.182	0.741	1.363	0.503–3.693	0.543	1.458	0.532–3.995	0.464
T3	37/1524	4.788	2.304–9.954	<0.001	4.974	2.377–10.406	<0.001	5.023	2.371–10.640	<0.001

Model 1: Original model

Model 2: Adjusted for age and sex

Model 3: Adjusted for age, sex, BMI, SBP, DBP, smoking, drinking, ACS, TC, and eGFR.

## Discussion

In this study, SHR was independently associated with in-hospital mortality in patients with CAD, especially in the pre-DM and DM group. Patients with the highest SHR (T3 group) had an increased risk of in-hospital mortality, regardless of their DM status. SHR and in-hospital mortality had a dose-response relationship, and the ORs for in-hospital mortality increased when the SHR index was above 1.20. In addition, the AUC of the SHR for predicting in-hospital deaths in patients with CAD was 0.741, which is interpreted a strong predictive ability.

The hypothalamic-pituitary-adrenal axis and sympathoadrenal system are activated and the release of proinflammatory cytokines is increased in response to stress [15]. These processes act synergistically to induce stress hyperglycemia [15]. Moderate stress hyperglycemia is a protective response to stress [16]. In an animal model of shock, external highly permeable glucose increased cardiac output and improved survival [17]. However, several studies have reported that stress hyperglycemia is strongly associated with adverse clinical outcomes among patients with critical illnesses, including myocardial infarction, stroke, sepsis, and COVID-19 [11, 18, 19]. Stress hyperglycemia has been identified as an independent risk factor for worsened prognoses in patients with ACS [4–6, 20]. Furthermore, accumulating evidence indicates that stress hyperglycemia was associated with a larger myocardial necrosis and poorer short and long-term prognosis in patients with myocardial infarction with non-obstructive coronary arteries (MINOCA) [21, 22]. The mechanism of this association may be related to a decrease in endothelium-dependent vasodilation, impaired platelet anti-aggregatory effects, and hyperactivation of sympathetic nerves with a proinflammatory pathway [23–25].

The threshold should be different in individuals with and without diabetes if the blood glucose level on admission is used to define stress hyperglycemia. Thus, stress-induced hyperglycemia cannot simply be represented by the ABG level but should be considered in conjunction with the status of chronic hyperglycemia. Robert et al. proposed the SHR index to represent relative hyperglycemia using the ratio of ABG to estimated chronic blood glucose [9]. Nathan et al. further suggested using HbA1c to estimate average glucose (AG) using the following equation: [AG (mg/dl) = 28.7 x HbA1c − 46.7] [26]. Several studies including patients with ACS have been conducted to verify the predictive value of SHR for adverse outcomes (Supplementary material Table S1). Marenzi et al. found that SHR was a more accurate predictor of in-hospital morbidity and mortality than ABG in patients with acute myocardial infarction [10]. Sia et al. found that SHR is an independent predictor of one-year mortality in patients with ST-segment elevation myocardial infarction (STEMI) with and without diabetes mellitus [27]. Xu et al. reported that SHR was significantly associated with 30-day mortality in patients with STEMI and that the predictive efficiency of the TIMI risk score can be improved after adding SHR as one point [12]. Yang et al. observed a J-shaped relationship between SHR and in-hospital cardiac death in patients with ACS who underwent the implantation of a drug-eluting stent [11]. Chen et al. reported a dose-response relationship between SHR and in-hospital deaths in patients > 75 years of age with acute myocardial infarction [28].

Consistent with these previous studies, the results of the current study indicate that SHR is significantly associated with in-hospital mortality in patients with CAD, especially in those with pre-DM and DM. Moreover, a dose-response association was observed between SHR and in-hospital mortality in the RCS analysis. A previous study reported a 7.9% mortality rate among patients with CAD, stable angina, and ACS who underwent percutaneous coronary intervention (PCI) over a median follow-up period of 2.5 years, suggesting that SHR is an effective marker of major cardiovascular and cerebrovascular events (MACCE) in patients after PCI, especially in patients with STEMI and without diabetes mellitus [29]. However, patients with the highest SHR in the current study (T3 group) had an increased risk of in-hospital mortality both in the ACS and CCS population. This difference may be attributed to different study populations. The proportion of patients with ACS in the previous study (48.3%) was higher than that in the current study (36.6%). In addition, in-hospital mortality within 30 days of admission was measured in the current study, which represents short-term mortality. As previously reported, SHR may not be effective in predicting long-term mortality [30]. This may be due to the fact that stress hyperglycemia represents only a transient stress-induced response during critical severe illness, while the factors affecting long-term mortality may be related to age and medical complications [30]. In the current study, the long-term mortality rate of patients with CAD was not measured. Therefore, the relationship between SHR and long-term mortality in patients with CAD should be validated in future studies.

### Strength and limitations

This study included a relatively large cohort of patients with CAD. This is the first report of a dose-response relationship between SHR and in-hospital mortality in patients with CAD using RCS analysis. However, this study is not without limitations. First, this was a single-center study and included only Asian patients. These results should be interpreted with caution. Second, the current study is limited by its retrospective design, and a causal relationship cannot be inferred in this study; further prospective multi-center studies are needed to validate these results. Third, this cohort study did not collect the information regarding hypoglycemic treatment of the participants; thus, further associations between SHR and the hypoglycemic treatment could not be evaluated.

## Conclusion

SHR is significantly associated with in-hospital mortality in patients with CAD. SHR may be an effective predictor of in-hospital mortality in patients with CAD, especially for those with pre-DM and DM.

## Electronic supplementary material

Below is the link to the electronic supplementary material.

Supplementary Material 1: Association of stress hyperglycemiaratioandin-hospital mortality in patients withcoronaryartery disease: Insights froma large cohortstudy

## Data Availability

The datasets generated and analysed during the current study are not publicly available due privacy and ethical restrictions but are available from the corresponding author on reasonable request.

## Abbreviations

ACS: acute coronary syndrome.
SHR: stress hyperglycemia ratio.
CAD: coronary artery disease.
OR: odds ratio.
CI: confidence interval.
DM: diabetes mellitus.
eGFR: estimated glomerular filtration rate.
SBP: systolic blood pressure.
DBP: diastolic blood pressure.
HR: heart rate.
TC: total cholesterol.
TG: triglycerides.
HDL-C: high-density lipoprotein cholesterol.
LDL-C: low-density lipoprotein cholesterol.
BMI: body mass index.
ABG: admission blood glucose.
NGR: normoglycemia.
SD: standard deviation.
RCS: restricted cubic splines.
ROC: receiver operating curves.
AUC: area under the curve.
CCS: chronic coronary syndromes.
MINOCA: myocardial infarction with no obstructive coronary atherosclerosis.
AG: average glucose.
STEMI: st-segment elevation myocardial infarction.
PCI: percutaneous coronary intervention.
MACCE: major cardiovascular and cerebrovascular events.
