# Supplementary material for: Association of stress hyperglycemia ratio and in-hospital mortality in patients with coronary artery disease: insights from a large cohort study
Source: Cardiovasc Diabetol. 2022 Oct 19;21:217. doi: 10.1186/s12933-022-01645-y (PMC9580448; doi:10.1186/s12933-022-01645-y)
Supplement: Supplementary file 1 — Supplementary Material 1: Association of stress hyperglycemiaratioandin-hospital mortality in patients withcoronaryartery disease: Insights froma large cohortstudy [file 12933_2022_1645_MOESM1_ESM.pdf]

Supplementary material

Association of stress hyperglycemia ratio and in-hospital mortality in patients with coronary artery disease: Insights from a large cohort study

Table S1 and Figure S1

Table S1. Description of the relevant studies in discussion section

AMI, acute myocaridal infarction; SHR, stress hyperglycemia ratio; CI, confidence interval; STEMI, ST-segment elevation myocardial infarction; NSTEMI, Non-ST-segment elevation myocardial infarction; MACE, major adverse cardiovascular events; MACCE, major adverse cardiovascular and cerebrovascular events; ACS, acute coronary syndrome; OR, odds ratio; CAD, coronary artery disease

Authors	Country	Year	Population	Endpoints	Key findings
Ma et al.	Italy	2018	1553 individuals with AMI	The composite end point of in-hospital mortality, acute pulmonary edema, and cardiogenic shock.	SHR showed the best prognostic power in predicting the end point as compared with glycemia at admission in the entire population (net reclassification improvement 12%[95%CI 4–20]; P= 0.003）
Sia et al.	Singapore	2021	5841 individuals with STEMI and 4105 individuals with NSTEMI	1- year all- cause mortality	In STEMI patients, SHR were independent predictors of 1- year all- cause mortality [OR 2.20 (95% CI 1.73–2.79)].
Xu et al.	China	2022	5417 individuals with STEMI	MACE and all-cause mortality in 30 days	SHR was significantly associated with 30-day mortality in patients with STEMI and that the predictive efficiency of the TIMI risk score can be improved after adding SHR as one point.
Yang et al.	China	2022	5562 individuals with ACS who undewent drug-eluting	MACCE and MACE at 2-year follow-up.	There were U-shaped associations of SHR with MACCE rate and MACE rate at 2-year follow-ups and J-shaped associations of SHR with in-hospital

			stent implantation		cardiac death and MI and that at 2-year follow-up in the study population.
Chen et al.	China	2021	341 individuals with AMI and aged over 75 years	MACCE and in-hospital mortality	Higher SHR was independently associated with in-hospital MACCEs ([OR]: 2.945, 95% confidence interval [CI]: 1.626–5.334, $P < 0.001$) and all-cause death (OR: 2.871 95% CI: 1.428–5.772, $P = 0.003$). This relationship increased with SHR levels based on a non-linear dose-response curve.
Yang et al.	Korea	2017	4362 individuals with CAD who underwent percutaneous coronary intervention	MACCE	Compared with the lower three quartiles of SHR, the HR (95% CI) for the highest SHR quartile (Q4) group for MACCE was 1.31 (1.05, 1.64) in the total population and 1.45 (1.02, 2.06) in the non-diabetic population.The risk of MACCE in the SHR Q4 group was significantly higher in patients presenting with STEMI.
Schmitz et al.	Germany	2021	2311 individuals with AMI	28-day mortality and 5-year all-cause mortality	Stress hyperglycemia plays a significant role mainly regarding short-term prognosis, in particular, in non-diabetic patients, but not for long-term prognosis.

Figure S1 (a) Area under the curve for admission blood glucose, HbA1c, stress hyperglycemia ratio (SHR) in total study population; (b) Area under the curve for admission blood glucose, HbA1c, stress hyperglycemia ratio (SHR) in patients with coronary artery diseases and diabetes; (c) Area under the curve for admission blood glucose, HbA1c, stress hyperglycemia ratio (SHR) in patients with chronic coronary syndromes; (d) Area under the curve for admission blood glucose, HbA1c, stress hyperglycemia ratio (SHR) in patients with acute coronary syndromes.

AUC, area under curve; CI, Confidence interval.

(a)

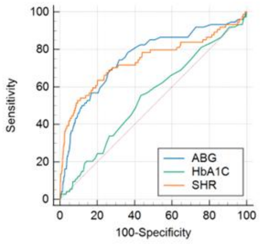

Variable	AUC	95% CI
ABG	0.757	0.747 - 0.766
HbA1C	0.543	0.532 - 0.554
SHR	0.741	0.732 - 0.751

(b)

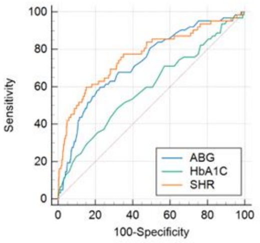

Variable	AUC	95% CI
ABG	0.728	0.715 - 0.741
HbA1C	0.594	0.579 - 0.609
SHR	0.766	0.754 - 0.779

(c)

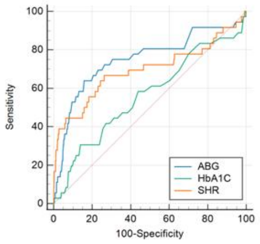

Variable	AUC	95% CI
ABG	0.750	0.738 - 0.762
HbA1C	0.563	0.549 - 0.576
SHR	0.694	0.681 - 0.706

(d)

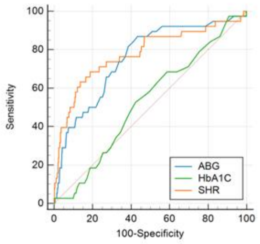

Variable	AUC	95% CI
ABG	0.757	0.741 - 0.772
HbA1C	0.528	0.510 - 0.546
SHR	0.779	0.763 - 0.793
